# Towards a Functional Account of Mass-Shooting: Prediction and Influence of Violent Behavior

**DOI:** 10.1007/s40614-025-00483-z

**Published:** 2025-11-24

**Authors:** James N. Meindl, Jonathan W. Ivy, Diana M. Delgado, Lindsey Swafford

**Affiliations:** 1 Applied Behavior Analysis, University of Memphis, 400B Ball Hall, Memphis, TN 38152 USA; 2 Psychology, Pennsylvania State University-Harrisburg, Harrisburg, PA USA

**Keywords:** Mass shooter, Behavior analysis, Intervention, Fame-seeking

## Abstract

Mass shootings affect both local and national communities and prompt extensive efforts to understand and prevent future events. Current approaches typically focus on profiling and typologizing mass shooters. Although these efforts are useful towards prediction of mass shootings, they do not tell us how to directly influence a shooter’s behavior. Thus, our understanding of mass shootings remains incomplete. Given that behavior analysis is a systematic natural science approach to understanding all behavior, we believe it is poised to address this issue. This article focuses on fame-seeking shooters, which are a subset of all mass shooters. We first describe important behavior patterns and contextual events that have been associated with this subset. We propose that these behaviors are members of a larger response class which includes a mass shooting. We then provide a conceptualization of the selection process involved in the emergence of mass shooting behavior and its precursors. We close by describing several interventions aimed at disrupting the contingencies identified by the conceptual analysis. The goal of this article is to illustrate how behavior analysis may utilize and extend the currently existing and predominantly non behavior-analytic research (e.g., profiling and typologizing based on the form of behavior) to better enable the prediction and influence of mass shootings.

A mass shooting violates social rules on a grand scale with devastating impacts that extend beyond the communities directly affected to reach national levels. Compared to other forms of lethal violence (e.g., gang or domestic violence), the seemingly random nature of a mass shooting can make an entire population feel vulnerable (Lowe & Galea, 2017). Although mass shootings are infrequent events that result in far fewer deaths each year relative to causes such as drug overdoses, car accidents, and accidental gunshots (Kane et al., 2023) mass shootings are unique in that the actions of one person affect so many.

Mass shootings are commonly defined as events that (a) result in the death of three or more people (excluding the offender); (b) are not familicide or criminal activity (e.g., not gang violence or armed robbery); and occur within one event in a public setting (Follman et al., 2025).

It is important to point out that this narrow definition excludes familicide and criminally motivated mass shootings that are sometimes included in overall tallies of mass shootings. Although this substantially reduces the number of incidents, it allows for clearer focus on public, often fame-seeking, mass shootings that may differ functionally from other forms of homicide in meaningful ways. Although mass shootings occur worldwide, they happen far more frequently in the United States than other developed or developing countries (Silva, 2022a). Using the definition provided above, there have been 152 such mass shootings in the United States between 1982 and 2024 (Follman et al., 2025) according to the *Mother Jones U.S. Mass Shootings* database (Follman et al., 2025). It is important to note that there has been an increasing trend in the number of mass shootings per year when adhering to this definition. Figure 1 displays yearly incidents of mass shootings in the United States during this timeframe

**Fig. 1:**
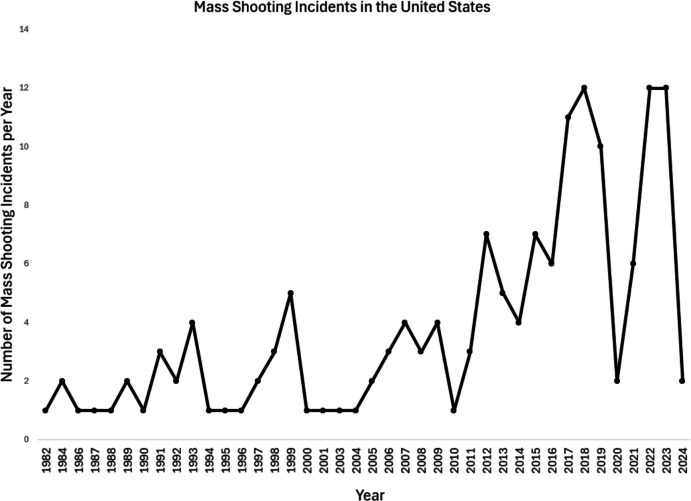
Mass Shooting Incidents per Year in the United States. *Note.* Data obtained from Follman et al., 2025

There may be multiple cultural and/or country-level differences that help explain variations in prevalence across countries. These may include the amount of media attention provided to mass shooters, differences in the availability of firearms, or variations in cultural practices that shape violence (Mattaini, 2003). Although these macro-level factors are important variables, a detailed cross-cultural analysis is beyond the scope of this article.

As a natural science framework for understanding all behavior (Skinner, 1974), behavior analysis takes a functional approach to explaining violent behavior. This approach assumes a selectionist process is involved where some forms of behavior are selected over others by the environment over the lifespan of the organism (Skinner, 1953). To identify variables responsible for this selection, behavior-analytic research relies on functional analysis methodology (Colombo et al., 2024; Iwata et al., 1982/1994), which typically requires a direct analysis of repeated incidents of the behavior of interest. Given that mass shootings are difficult to predict and occur only one time as the shooter is either arrested or dies during the event (Lankford, 2016), a repeated analysis of mass shooting may be difficult. It may be possible, however, to provide a behavior-analytic conceptualization of a mass shooting. Such an account may help identify functional relations between relevant behavior and environmental variables and direct future empirical efforts at intervention development. In this approach, behavior patterns are identified and variables responsible for the selection and maintenance of these behaviors are suggested.

Previous research has extended behavior analysis to violence (e.g., Sturmey, 2022) and largely focuses on collective violence and cultural shaping processes. For example, Mattaini (2003) highlights the importance of a functional approach to collective violence and emphasizes the need to develop culture-level nonviolent functionally equivalent alternatives. Mattaini and Strickland (2006) suggest a natural science approach to collective violence and emphasize the operant processes, cultural practices, and interlocking contingencies that help shape and maintain group level violence (e.g., terrorism, insurgency). Roose and Mattaini (2020) discuss different types of violence (interpersonal, collective, structural) and situate mass shooting within a broader ecological framework. They then outline a constructive program to build alternative ecologies. This article seeks to build on this overall line of behavior-analytic research on violence. Whereas previous accounts have largely focused on collective forms of violence and the interlocking contingencies and ecologies shaping the behavior, this article provides a functional account of mass shooting at the level of the individual. To our knowledge no such accounts of mass shooting have been put forth.

In this article we begin by describing the general traditional approaches to understanding mass shootings, describe the limitations of such approaches, and detail the benefits of a functional strategy. We then provide a conceptualization of the selection of mass shooting behavior and its precursors. We focus our analysis on “fame-seeking” mass shooters (described below; Lankford, 2016). Fame-seeking mass shooters were selected as a starting point because they have become an area of increased research, and the associated precursor behavior patterns are relatively well-documented (Silva & Greene-Colozzi, 2019; Silva et al., 2023; Succar et al., 2023). Further, the incidents of mass shootings motivated by fame appear to constitute a growing proportion of overall mass shootings (Lankford, 2016; Silva, 2022b). Finally, relative to other mass shooter types, which are categorized topographically, fame-seeking mass shooters are generally functionally defined.

In our analysis we describe general behavior patterns and contextual events that have been identified with fame-seeking mass shooters. We then provide a conceptual analysis of these behavior patterns and contextual events, explain how they create behavior traps (Baer & Wolf, 1970) that select increasingly restricted response classes, and describe how these events contribute to or influence a mass shooting. Finally, we describe interventions suggested by the resulting analysis and detail how these interventions could be implemented as primary or secondary prevention of mass shooting. It is important to note that mass shooters are not a monolith, and this article does not purport to provide a unifying account applicable to all mass shooters or even all fame-seeking shooters. Although a functional approach could be applied to all mass shooters, the individual resulting analyses would differ as it is individual genetic endowment, learning histories, and cultural variables that influence behavior (Skinner, 1981).

## Limitations of Current Approaches to Understanding Mass Shootings

The typical response by the lay public, media, and politicians to a mass shooting is to focus blame on past traumas or mental illness experienced by the shooter (Hirschtritt & Binder, 2018), to renew debates of gun control versus gun rights (Metzl & MacLeish, 2015), or to enact policies aimed at strengthening security (Jonson, 2017). Other professionals (e.g., administrators, social workers) may enact preventative measures such as staff trainings, bullying prevention programs, threat/risk assessments, trauma screenings, and enhanced security policies (Calhoun & Weston, 2015; Dorris & Murphy, 2023; Gregory & Park, 2022). Researchers in the area of mass shootings have largely focused on developing mass shooter typologies (e.g., pseudocommando, set-and-run killer; Dietz, 1986), categories (e.g., disgruntled employee versus rampage shooter; Capellan et al., 2019), and profiles (e.g., young, male, white; Capellan & Gomez, 2016), or analyzing the temporal relation that one mass shooting has with another (Towers et al., 2015). Although many of these efforts may minimize injury (e.g., through security hardening) or identify correlates that support some *prediction* of a mass shooting (e.g., younger people are more likely to commit a mass shooting), they do not specify manipulable environmental variables in a functional relation with mass shooting behavior and thus do not suggest ways to directly *influence* the behavior.

Identifying functional relations that enable both the prediction and influence of behavior is a primary goal of behavior analysis (Skinner, 1953; Watson, 1913). Identified causes of behavior must be both manipulable (in principle) and located in the environment rather than additional behavior of the individual (Skinner, 1953). These causes must begin and end beyond the person’s own behavior because behavior itself is a dependent variable (Hayes & Brownstein, 1986). A sufficient accounting of behavior, therefore, identifies *environment*-behavior functional relations rather than ending with descriptions of behavior-behavior relations. Given that behavior is a function of environmental/contextual variables, the presence or absence of these variables helps predict behavior. As these variables are physical and manipulable events, they may be acted upon to influence occurrences of the behavior. This is true regardless of whether the behavior is publicly observable or private and within the skin of the individual (Meindl & Ivy, 2023). Behavior–behavior relations may enable prediction of behavior but do not enable the influence of behavior, which can only be changed by manipulating the environmental variables in a functional relation with that behavior.

Many of the current efforts by the public, professionals, and researchers appear to primarily suggest behavior–behavior relations. To the extent mental illness contributes to a mass shooting (and that extent is debatable; see Metzl & MacLeish, 2015) it represents a behavior–behavior relation (i.e., mental illness—understood as a descriptive term that involves certain behaviors—causes mass shooting) and thus does not suggest ways to directly influence mass shooting behavior. As an example, a person diagnosed with a social anxiety disorder may avoid crowds, but suggesting the disorder *caused* the avoidance (i.e., a behavior–behavior relation) would be insufficient for the purposes of influence. Social anxiety disorder is neither distinct from the avoidance of crowds (it is a label of the avoidance), nor can it be directly manipulated. An analysis of environment–behavior relations might suggest avoidance occurs because the presence of large groups of people is aversive, and this aversive condition is removed through avoidance behavior. This analysis suggests directly manipulable variables (i.e., presence and removal of people) and suggests ways to influence avoidance behavior, such as providing reinforcement for approach and extinction for avoidance behavior (e.g., Harper et al., 2013).

Efforts in developing typologies, categories, and profiles suffer from similar limitations. These efforts may prove useful in developing general predictions (e.g., future mass shootings are likely to be perpetrated by young white males) and may help identify patterns of behavior shared by multiple mass shooters (e.g., patterns of planning and preparation). However, these efforts do not identify environment–behavior functional relations and thus do not specify manipulable causal variables, do not tell us why the identified behaviors occurred, and do not suggest ways to directly influence the behavior.

Finally, although the prevalence of guns may be associated with differing rates of violence, and highly secure buildings may deter shooters to other locations, at best these analyses suggest ways to manipulate response effort or eliminate opportunities for behavior to occur. No insight is provided into the learning history leading to the event. As an example, we might note that a person engages in less aggression when handcuffed, but such an analysis tells us nothing about how aggressive behavior was learned, why it occurs, or how to alter the likelihood of aggression in the future should opportunity arise.

## Extending Behavior Analysis to Fame-Seeking Mass Shooter Behavior

The behavior-analytic approach of identifying environment-behavior relations has been extended to better explain a range of socially significant behaviors such as terrorism (Dixon et al., 2003), depression (Dougher & Hackbert, 1994), attention deficit hyperactive disorder (Mitchell, 2009), flirtation (Wade, 2018), theory of mind (Degli Espinosa, 2025), dreaming (Dixon & Hayes, 1999), intimacy (Cordova & Scott, 2001), creativity (Amezquita, 2021), vandalism (Mayer et al., 1993), gambling (Dixon et al., 2015), sexual abuse (Lumley et al., 1998), and language and cognition (Barnes-Holmes et al., 2017).

In each case, analysis seeks to suggest contingencies involving antecedent and consequence variables that evoke and strengthen the behavior. Assessing immediate contingencies helps identify contextual variables experienced on an ongoing basis including motivational states, consequences directly affecting different behaviors, and contextual events influencing behavior probabilities. For example, an analysis of a child’s violent tantrums may find that tantrums are evoked when the child is denied a toy and strengthened by provision of that toy contingent on tantrums. Similar analytic strategies can be extended to more complex behaviors such as terrorism or mass shootings, where immediate contingencies likewise shape and maintain responding.

Particularly with organisms that engage in verbal behavior, rules and relational responding may influence the effectiveness of immediate contingencies in selecting behavior and allow individuals to respond to a range of complex contingencies. Rules are verbal statements about contingencies and can change the function of stimuli within the contingency (Blakely & Schlinger, 1987). Relational responding is responding to one contextual event based on how it relates to another (e.g., same versus different, more than versus less than, my perspective versus your perspective). When several relations between stimuli are established, additional relations and increasingly complex rules may be derived, and the function of stimuli in the relations may be transformed by the nature of the relations (Barnes-Holmes et al., 2017; Hayes et al., 2001). For example, if someone is taught that killing a person will result in media attention, and that not killing a person will result in no media attention, a relation may be derived that the more people they kill the more media attention they will receive. If attention is valuable, more deaths would become a more powerful outcome than fewer deaths. A detailed discussion of rules and derived relational responding falls outside the focus of this article, but see Stewart et al. (2013) and Harte et al. (2020) for an overview.

Dixon et al. (2003) illustrate the potential interconnection between these direct contingencies, rules, and derived relational responding in their hypothetical analysis of a potential terrorist’s radicalization. In brief, they describe a man who directly experiences a contingency between poor economic affairs and a lack of food. He learns a rule specifying a contingency between lack of food and the actions of “America,” and that America is also evil. He then learns that a local terrorist group is the opposite of—and fights against—America. A new relation may now be derived: the terrorist group is good, and fighting America is good, because America is evil and the organization is the opposite of America. As Dixon et al. outline, “a member of [this terrorist group] need not directly experience martyrdom for that outcome to be reinforcing. It is enough that the act of martyrdom itself participates in a whole host of relations with other verbal events, self-statements, and derived consequences” (p. 136).

The concept of a response class is also essential to explaining and understanding complex behavior. Many behaviors exhibited by an individual may be influenced by the same functional variables, and thus are said to be part of the same response *class,* even if they look topographically dissimilar and vary in relative probabilities. It is important to note that intervention targeting one member of a response class may produce changes in other members of the same class (Lalli et al., 1995; Parrish et al., 1986; Sprague & Horner, 1992).

A mass shooting may be understood as one extremely problematic member of a response class. Other behaviors may share common membership in this response class, such as the precursor behavior patterns identified in the behavior–behavior relations involving mass shootings (described below). Whereas a mass shooting occurs only once, these other response class members have the benefit of being more readily analyzed. For example, a behavior–behavior relation may be found between verbal threats of violence and subsequent mass shootings, suggesting both share membership in the same response class. Given verbal threats happen at a much higher probability than a mass shooting, an analysis of these verbal threats (or other higher probability functionally equivalent responses) may be possible. Subsequent intervention to decrease verbal threats may similarly influence the likelihood of a mass shooting.

**Fig. 2:**
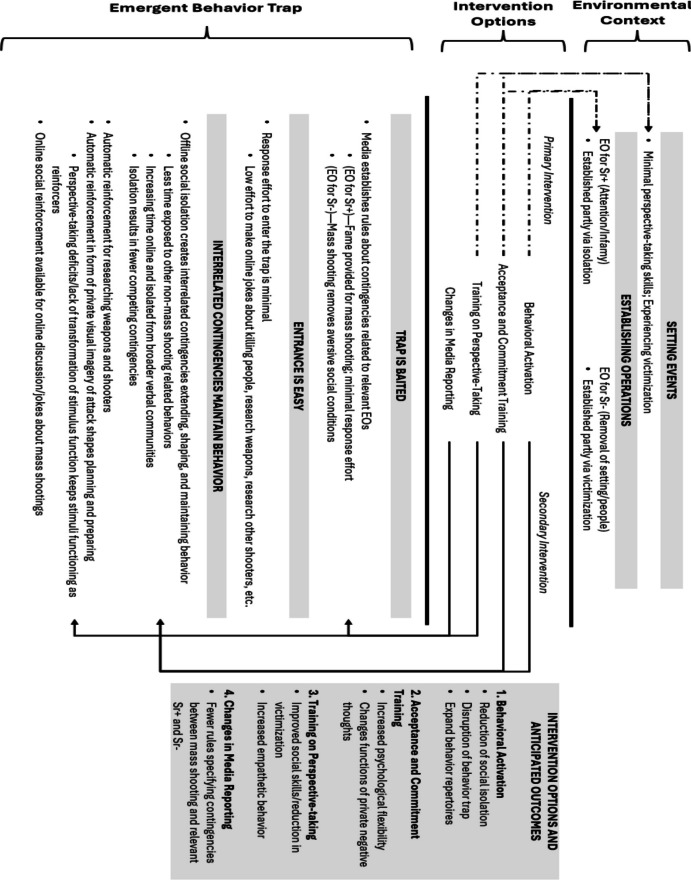
Describes Setting Events, Establishing Operations, Emergent Behavior Trap, and Associated Interventions. *Note.* Dashed arrows indicate primary intervention targets. Solid arrows indicate secondary intervention targets. The shaded Intervention Options box describes intended intervention outcomes

Information on current contingencies helps identify how relevant behavior is maintained and suggests immediately manipulable variables. Information on rules and derived relational responding repertoires suggests skill deficits that may affect those contingencies and suggests instructional strategies to build relevant skills. Suggested shared membership in a response class broadens the range of behavior that may be analyzed. What follows is an analysis of the behavior of a fame-seeking mass shooter explaining how currently identified behavior patterns and contextual variables influence a mass shooting. Figure 2 outlines these behavior patterns and contextual variables, an emergent behavior trap, and indicated interventions.

## A Functional Account of Fame-Seeking Mass Shooter Behavior

Infamy, glory, or attention appear to be a potent reinforcer for fame-seeking mass shooters (Lankford, 2016). This is inferred by the shooter’s behaviors,^1^ which include either directly stating this motivation, leaving behind legacy tokens (e.g., video statements), or posting material online shortly before the shooting. Compared to other mass shooters, fame-seeking shooters tend to be younger (average of 20.4 vs. 34.5 years old) and produce higher death counts (average of 7.2 vs. 3.0 deaths per incident; Lankford, 2016). In addition, they are more likely to be diagnosed with a clinical disorder, have a history of engaging in behavior associated with suicide, engage in grandiose behavior (Lankford, 2016), and display “narcissistic” behavior patterns (Bushman, 2018). The analysis outlined here will focus on several specific behavior patterns and contextual variables associated with fame-seeking mass shooters. These include: (a) motivations for fame/infamy (Lankford, 2016); (b) a lack of empathetic behavior and poor perspective-taking skills (Bushman, 2018; Peterson et al., 2024); (c) victimization (Silva & Greene-Colozzi, 2019); and social isolation (Peterson et al., 2024; Silver & Silva, 2022).

### Fame, Infamy, and Attention

Fame/infamy and attention are more powerful putative reinforcers for fame-seeking shooters relative to other shooters or the public (Bushman, 2018; Lankford, 2016). Activities that produce high magnitudes of fame (e.g., professional athlete, career musician), however, are often quite effortful and the behaviors associated with these activities are far from guaranteed to produce fame. As a result, strong motivation exists for fame but there are few opportunities to produce these consequences in large magnitudes.

A potential mass shooter motivated by fame or attention can learn rules specifying contingencies between mass shooting and fame production through various media sources (Meindl & Ivy, 2017, 2018). They may learn that a mass shooting is nearly guaranteed to produce fame and that this attention will be immediate, significant, and long-lasting (Silva & Greene-Colozzi, 2019). Additional rules specify that there are minimal barriers to engaging in mass shooting as it is relatively low effort, does not require thousands of hours of practice to perform effectively, does not require significant talent or skill, and is relatively inexpensive. Finally, the potential shooter learns that higher victim counts produce higher levels of fame, and that a shooting is an opportunity to leave a manifesto that may further heighten the fame and afford the recognition the shooter believes is deserved (Langman, 2017).

Existing research suggests a link between media-provided attention and mass shootings. Studies on contagion effects indicate that when a mass shooting is highly publicized there is an increased likelihood of another mass shooting in the next several weeks (Pescara-Kovach & Raleigh, 2017; Towers et al., 2015). This potential for the provision of fame is the impetus for various campaigns such as “No Notoriety” and “Don’t Name Them,” which urge media outlets to withhold the names and images of mass shooters (Lankford & Madfis, 2018).

### Empathetic Behavior and Perspective-Taking

Deficits in empathetic behavior are a characteristic of mass shooters in general (Bushman, 2018; Peterson et al., 2024). Although various conceptualizations of empathy exist (Batson, 2009; Hall & Schwartz, 2019), the term generally describes the ability to perceive and experience the emotions of others (Cuff et al., 2016; Taylor et al., 2018). A foundational prerequisite of empathetic behavior is perspective-taking (Vilardaga, 2009), and the better someone is at discerning the behavior and motivation of others, the more empathetic behavior that person generally displays (Eisenberg, 2000). Empathy is not a quality within a person—it is a behavior that requires the prerequisite skill of perspective taking (Kavanagh et al., 2020).

Perspective taking is an instance of relational responding wherein a person can respond to one context (their perspective) in terms of another context (someone else’s perspective; Barnes-Holmes et al., 2001). This is also referred to as deictic relational responding, is generally learned in early-mid childhood, and is well-established in typically developed adults (McHugh et al., 2004). A person with a strong repertoire can effectively discriminate the thoughts and feelings of others, act more empathetically, and their social life improves (Eisenberg, 2000). The worse someone is at this, the less they modulate their emotions towards others, which often leads to avoidance of social settings, decreased social interactions, and fewer empathetic behaviors.

Deficits in deictic relational responding have been associated with a range of disorders including autism (Rehfeldt et al., 2007), schizophrenia (Villatte et al., 2010), depression and social anhedonia (Villatte et al., 2008), and social anxiety disorder (Janssen et al., 2014). Understanding perspective taking as an instance of relational responding is beneficial as it specifies a learned behavior (rather than an innate ability; e.g., Baron-Cohen, 1997) that is amenable to influence (Barnes-Holmes et al., 2004; Degli Espinosa, 2025) and helps explain the relation between perspective-taking and empathetic behavior. A person with a strong perspective-taking repertoire can both describe how someone else is feeling (i.e., tact their controlling variables, Degli Espinosa, 2025) and may also experience some of those feelings directly through the transformation of stimulus function (Dymond & Rehfeldt, 2000).

Deficits in perspective taking may be inferred in mass shooters given the minimal empathetic behaviors displayed. If a person has difficulty taking the perspectives of others, some social controls of behavior would not be expected to fully emerge. Although the individual may accurately describe how their behavior will affect others, they would have difficulty assuming the perspective of the victims (i.e., responding relationally). When the potential shooter imagines the shooting, they view the imagined context from their own perspective only and would also “feel” from their own perspective only (i.e., there would be no expected transformation of stimulus function). Rather, the predominant experience would be the reinforcing aspects of the shooting (e.g., fame/infamy, removal of aversive stimuli and people).

### Victimization

Fame-seeking mass shooters are more likely to experience victimization (physical, emotional, or sexual) than other mass shooters (Silva & Greene-Colozzi, 2019). As a contextual variable, victimization can create powerful and pervasive aversive conditions (Vidourek et al., 2016). When an individual is bullied at school, for example, the actual physical or emotional interaction itself is aversive, and the bully may acquire aversive properties. This aversive conditioning can then generalize beyond the bully to other classmates, teachers, or the school itself. Thus, an individual who experiences victimization at school may find an entire school environment aversive even though it is only the act of one bully that produced the immediate aversive stimulation. This same generalization process would apply to many victimization contexts, such as a work setting acquiring aversive properties for someone victimized at work. Further, if someone is victimized in many locations the aversive properties would be expected to generalize more broadly.

When an aversive condition is in place, people generally engage in behaviors that remove those aversive conditions (Catania, 2013). This removal may take a variety of forms but can be broken into categories of behavior that escape from or avoid the aversive event. Although both behaviors remove the aversive condition, the behavioral topographies associated with the two categories may look quite different. When the aversive condition is related to victimization at school, for example, avoidance behavior may take the form of ceasing attendance or changing schedules to avoid certain people. Under the same conditions, escape behavior may range from telling a teacher about the victimization, having the bully removed, or directly causing harm to the bully or other stimuli to which aversive properties have generalized. Thus, whereas avoidance behavior involves the person removing themselves, termination behaviors involve removing the aversive stimuli from the environment. It is the latter termination behavior that is partly on display when, for example, a person is victimized in a school environment and then returns to shoot classmates.

### Social Isolation

Social isolation is a common experience of perpetrators of mass shootings, particularly fame-seeking shooters (Peterson et al., 2024; Silva & Silva, 2022; Twenge & Campbell, 2003). Social isolation may be conceptualized as a context that both results in, and is the product of, a restriction of opportunities for positive social experiences or reinforcers. Behavior is controlled by the context, and behaviors that do not result in reinforcing outcomes are less likely to occur in the future (Lattal et al., 2013). If a person receives minimal reinforcement for engaging in social behaviors in certain contexts, as is the case for socially marginalized individuals, that person may engage in fewer social behaviors in those contexts. This results in fewer opportunities for social reinforcement and may exacerbate the decline in social behaviors. Thus, a decrease in diffuse sources of social reinforcement can lead to a decrease in social behaviors, which further decreases opportunities for social reinforcement and results in general social isolation.

In addition to changes in reinforcement contingencies, social isolation may also function as a motivating operation that establishes attention of various kinds (e.g., infamy, notoriety), and escape from aversive social contexts, as powerful reinforcers. Behaviors that lead to social isolation may be negatively reinforced by avoiding social interactions that have become aversive. However, social and nonsocial reinforcers are often available in other contexts and behavior may increase in those settings. A socially isolated individual may begin spending more time on the internet, for example, gravitating to websites that provide reinforcement (social or non-social) which has become more potent due to general isolation. If there is already some motivation for the removal of aversive stimuli as described above, these may be websites that actively reinforce discussion and humor around mass shootings. For example, in 2019 at least three mass shooters (Christchurch Mosque, El Paso Walmart, Chabad of Poway Synagogue) posted mass shooting related content to the online messaging board 8chan (Mezzofiore & O’Sullivan, 2019). 8chan was an anonymous messaging board that was particularly known for white supremacist, neo-Nazi, racist, and antisemitic content. Most of the site’s board postings reinforced extremely dark content and humor. The Christchurch Mosque shooter, for example, posted content and a link to a 17-min livestream of the shooting to 8chan, which was reshared and applauded by 8chan members. The Chabad of Poway Synagogue shooter explicitly cited 8chan as a radicalizing force in his actions (Dickson, 2019). Given online sources of social reinforcers for internet related behaviors, and few sources of social reinforcers for noninternet-related behavior for someone who is socially isolated, it would be unsurprising to find a person spending an increasing amount of time online. Given websites such as 8chan that explicitly reinforce posting extreme dark content, it would also be unsurprising to find this verbal behavior to increase as well for certain individuals. In essence, general social isolation can result in gravitation a highly insular online verbal community that is largely defined by a restricted interest in, and reinforcement of, violent and extreme verbal and nonverbal behavior.

Many nonsocial automatic reinforcers exist online and offline as well. Consider that 77% of mass shooters spent a week or longer planning their attack (e.g., decision making, site/target selection), and 46% of mass shooters spent a week or longer preparing for the attack (e.g., procuring firearm and ammunition; Silver et al., 2018). Given that these activities are done in isolation, this reinforcement is likely automatic, and the nature of social isolation means there are fewer opportunities for non-mass-shooting–related behaviors to be selected. For example, stimuli produced by planning or solving logistical problems (e.g., deciding how to enter a site, mapping routes) may itself be reinforcing, independent of any immediate social consequences. Thus, for certain individuals, social isolation may increase the probability of mass shooting planning and preparing behaviors—the decrease in diffuse sources of social reinforcers increases the probability of behaviors that result in nonsocial reinforcers.

### An Emergent Behavior Trap

Behavior traps are characterized by powerful reinforcers, low barriers to entry, and sets of interrelated contingencies that increase and maintain the behaviors associated with the behavior trap (Baer & Wolf, 1970). Behavior traps are frequently programmed to great effect to encourage and maintain academic behavior (e.g., Alber-Morgan & Heward, 1996) but can also help better understand a variety of social behaviors. An example of a behavior trap applied to social behaviors might be one of joining a church. There are powerful reinforcers (e.g., social interaction, promise of eternal salvation in some religions), low barriers to entry (e.g., you merely need walk in the doors), and various sets of interrelated contingencies maintaining further church attendance (e.g., people asking you to return, welcoming you when you come).

In the case of fame-seeking mass shooters a behavior trap may also emerge. First, there are heightened motivations for two specific reinforcers—fame/attention and the removal of the aversive social conditions (e.g., the people and contexts associated with victimization). A mass shooting directly produces these powerful reinforcers, which, if not directly experienced, is nevertheless learned via rules. Second, there is a low barrier to entry in that no particular skill is necessary to engage in the behavior and the necessary guns are easily obtained in the United States. Finally, there are interrelated contingencies maintaining the precursor behaviors leading up to the mass shooting. Researching different types of guns puts the potential shooter in contact with the direct and automatic reinforcement of finding the “right” weapon. Planning the shooting puts the shooter in contact with private visual imagery that strengthens planning behavior. These contingencies are strengthened by few competing reinforcers due to social isolation. Further, a deficit in perspective-taking/empathetic behavior means the stimuli produced by private visual imagining can function as reinforcers (see Meindl & Ivy, 2023, for a discussion on distinguishing private behaviors and private stimuli). Ultimately, the lack of reinforcers to redirect behaviors elsewhere results in an increasingly restricted set of behaviors associated with mass shooting.

## Intervening to Prevent Mass Shooting Behavior

The preceding analysis identifies immediate contingencies can select members of a response class that includes mass shooting. Further, skill deficits are suggested that allow those contingencies to be effective at strengthening and maintaining such behavior. This analysis posits manipulable maintaining variables suggestive of ways to influence mass shooting behavior. In particular, it is important to (a) consider dissemination of rules regarding mass shootings and attention; (b) focus on dismantling behavior traps; (c) teach better ways to respond to isolation and victimization; and increase perspective-taking/empathetic skills. Several interventions are briefly described below.

It is important to reduce the role media sources play in disseminating rules specifying a relation between mass shootings and the production of attention. Calls to withhold the names and faces of mass shooters from wide distribution currently exist (e.g., Lankford & Madfis, 2018). Further guidelines for reporting mass shootings such as not widely disseminating manifestos or writings, avoiding the suggestion a mass shooting is a means of retaliating against victimizers, minimizing sensational language and overall coverage is also available (Meindl & Ivy, 2018). It may also be possible to disseminate new rules by linking the behavior with undesirable qualities, such as highlighting the fact that many mass shooters also engage in domestic abuse (Krouse & Richardson, 2015). Similar efforts have been successful in other contexts, such as antismoking campaigns (e.g., Davis et al., 2018). Adopting this strategy would require careful attention to identify only qualities found objectionable by the target audience (i.e., potential mass shooters). Though currently proposed, it is unclear the extent to which reporting recommendations have been adopted. Further, it would be important to establish monitoring systems to determine the effects of any new rule dissemination.

For issues of social isolation and behavior traps, behavioral activation (Addis & Martell, 2004; Hopko & Lejuez, 2008; Hopko et al., 2003) may be indicated. The general components of behavioral activation include having the client (a) identify activities that are associated with positive feelings; (b) set small goals that expose them to those activities; (c) self-record engagement; and (d) gradually expanding the program to include longer term goals and increase the overall frequency of contact with pleasant activities.

The intervention is effective at decreasing depressive behaviors and social isolation and increasing contact with an array of reinforcers (Sturmey, 2009). A major focus of the intervention would be to promote contact with reinforcement in noninternet social environments, thereby decreasing contact with sources of reinforcement for mass shooting related behaviors and “pulling” behavior in different and more positive directions.

Acceptance and commitment training (ACT; Zettle & Hayes, 1986) may be beneficial for concerns regarding both social isolation and victimization. ACT is an effective intervention for a range of behavioral concerns related to negative thinking (e.g., “Everyone here hates me”) and has been shown to decrease experiential avoidance and enhance subjective wellbeing in both clinical and nonclinical populations (Hayes et al, 2004; Stenhoff et al., 2020). The intervention broadly aims to strengthen and promote psychological flexibility, which may be defined as the ability to fully contact the present moment and the thoughts and feelings it contains, and to align behaviors with one’s goals and values (Hayes et al., 2004). This intervention is effective in changing behavior across a range of conditions including depression, borderline personality disorder, generalized anxiety disorder, work related stress, psychosis, eating disorders, substance abuse, and other problems (Bach & Hayes, 2002; Bond & Bunce, 2000; Dalrymple & Herbert, 2007; Hayes et al., 2004). Further, there is emerging evidence that increases in psychological flexibility can have a beneficial impact on aggression and violent behavior (Berkout et al., 2019).

ACT directly teaches individuals to notice negative thoughts and experiences without judgement, to view those thoughts and experiences in context, and to let them pass without attempts to control or avoid them. For example, a person may have the thought, “everyone at school is rotten, and nobody likes me, and the world would be better without them.” They may also privately tell themselves, “I have nothing to live for; I might as well be the one to take them out!” Rather than attempting to directly challenge those thoughts (as might be the approach in cognitive behavior therapy [CBT]; Beck et al., 1987), ACT would teach the individual to recognize those thoughts and to view them in context—to create psychological “distance.” A goal would be to decrease the extent to which private verbal thoughts functioned as rules; the thought is merely a thought, not a command to be acted upon. A further goal may be to teach the individual that the large source of the overall aversive experience is not the actual people at school but rather the constant perseverative thinking about them, and it is this thinking that is preventing the person from moving in a direction they value. The person would be assisted in identifying those values and developing strategies to align behavior with them. The overall goal would be to increase psychological flexibility and subjective well-being (Stenhoff et al., 2020), decrease social isolation through decreased experiential avoidance, and promote a different relationship with victimization.

Finally, a deficit in empathetic behavior could be directly addressed by teaching perspective taking. This skill set has been successfully taught to individuals with schizophrenia (O’Neill & Weil, 2014), autism (Jackson et al., 2014), and young children (Weil et al., 2011), which may increase empathetic behavior toward outgroup members and decrease the fundamental attribution error (Hooper et al., 2015) where a person views their own mistakes as resulting from context, but the mistakes of others as resulting from internal dispositions.

### Primary and Secondary Prevention Efforts

These interventions could be implemented as primary or secondary prevention efforts. Primary prevention seeks to prevent the target behavior from ever developing, whereas secondary prevention emphasizes early detection and intervention. In the area of medicine, a primary prevention might be immunization whereas secondary prevention might be screening for infection after a potential exposure. In the area of behavior change, a schoolwide intervention would be an example of primary prevention, whereas targeted interventions for “at-risk” individuals might be secondary interventions (Tobin & Sugai, 2005). This prevention approach aligns with the three-tier model of positive behavior support (Sugai & Horner, 2002) although consideration of the third tier (tertiary support) may not be necessary given a mass shooting generally ends with the shooter’s death or incarceration.

We consider entry into the behavior trap to be an inflection point where targeted secondary intervention may be necessary. The nature of the behavior trap makes changing the trajectory of behavior more difficult and less likely to occur without outside intervention. Additional warning signs (described below) can be used to better identify “at-risk” individuals in need of targeted intervention. It is important to note that meaningful impact is most likely when primary and secondary interventions are implemented together, targeting individual skills training and broader interlocking cultural practices (Mattaini & McGuire, 2006).

### Primary Prevention

It may be possible to decrease mass shootings by preventing behavior patterns and experiences such as social isolation, motivations for infamy, deficits in empathetic behaviors, and motivations stemming from victimization from emerging in the first place. Given the described interventions would be widely applied, and that the targeted skills are arguably important for everyone, there is an added potential benefit of broadly increasing general well-being and decreasing other antisocial behavior. Further, these interventions are relatively quick to implement.

Implementing primary intervention for mass shooting would involve scaling interventions up and out. Scaling-up shifts the focus from the individual to the whole population with a focus on prevention. Scaling-out applies these interventions across a range of societal sectors such as schools, families, and community settings (Biglan et al., 2020). These efforts would be strengthened by action and implementation at a variety of levels such as governmental, media, local organizing, and changes in public policy (Biglan et al., 2012). Biglan (2009) suggests that efforts to scale interventions up and out typically start at the local level, and that positive outcomes help drive further adoption and spread.

The interventions suggested in this article can be scaled up and out. Acceptance and commitment training has been embedded as primary intervention already (Biglan, 2009) in programs such as the AIM (Accept. Identify. Move) Curriculum (Dixon & Paliliunas, 2018) which is a formalized set of ACT-based activities designed for school-aged students and largely implemented school-wide. Group behavioral activation, implemented as a primary prevention, has been shown to be equally or more effective than CBT at preventing depression (Biglan et al., 2008), and is considered both feasible and acceptable by group members (Belanger et al., 2025). Further, early studies have found that behavioral activation delivered via a self-guided mobile app may also be effective at preventing depression (Bralee et al., 2025; Edge et al., 2023), which is highly cost effective. Finally, many prevention programs targeting bullying (Rock et al., 2004), radicalization (Sklad et al., 2022), and improving intergroup attitudes (Beelmann & Heinemann, 2014) already include some instruction on perspective-taking and empathetic behavior. Behavior-analytically informed training could be embedded into such programs already being implemented at large-scale. Reforms in media reporting of mass shooting would be considered primary intervention as the many media sources (e.g., legacy, new, social) already contact broad audiences.

### Secondary Prevention

Secondary intervention requires identifying “at-risk” individuals, which may prove challenging with mass shooting behavior. Many of the contextual events and behavioral patterns described in this analysis are experienced or emitted by many individuals, yet only a tiny fraction will engage in a mass shooting. There are, however, indicators to help identify those in need of intervention. Behavior concerns are often evidenced years before a mass shooting (Lankford & Silva, 2021) and preattack warning signs are frequently present shortly before the event (Slemaker, 2023). Additional personal characteristics, such as expressed feelings of isolation or marginalization, hypersensitivity to perceived insults, a history of problematic behavior driven by acquiring fame, expressed admiration of individuals (particularly other mass shooters) who have responded to victimization with violence, fixation on the need for recognition, insistence that their problems are the result of the actions of others, and preoccupations with the meaninglessness of life are further warning signs (Lankford, 2018).

Perhaps the most critical warning sign is communication to a third party that an attack is imminent, known as leakage (Meloy & O’Toole, 2011). This could be in the form of a joke or threat about violence, suggestions of upcoming fame or going out in a “blaze of glory,” or planning or preparation indicators such as acquiring weapons, preparing legacy tokens, or engaging in reconnaissance (Lankford, 2018). A study of active shooters conducted by the Federal Bureau of Investigation (FBI) found that approximately 56% openly indicated intent or expressed specific violent thoughts prior to an attack (Silver et al., 2018).

The FBI suggests that when a threat is made, additional factors be considered such as motivation (e.g., is the intention to intimidate or punish others, protect others or oneself, respond to injustice), directness of the threat (e.g., direct, indirect, veiled, conditional), specificity (e.g., weapon, date, location), emotional content of the threat, and the existence of any precipitating stressors (O’Toole, 2000). Low risk threats are vague, indirect, include minimal details, and appear unlikely given the content. Medium level threats are more direct but are implausible or inconsistent; some details are provided but there is no indication any preparation or planning has taken place. High level threats are direct, specific, credible, plausible, and include indications that preparatory steps have taken place (O’Toole, 2000).

It may be possible to better identify those in need of secondary intervention by considering personal characteristics, specific warning signs, leakage about an attack, and medium to high level threats. False positives may be minimized by incorporating interobserver agreement reliability checks when assessing “at-risk” individuals. Early identification in schools may be supported by teacher and parent training designed to improve recognition of early warning signs. Although it may be more difficult to identify potential shooters outside of a school setting, raising awareness of warning signs across community contexts could support identification. In both contexts, appropriate referral processes should be emphasized. Once an individual is identified as “at-risk” it may be possible for trained individuals to conduct more specific analyses to determine relevant contextual events and behavior patterns and to implement targeted secondary intervention.

Whether implementing primary or secondary intervention, a monitoring system should be incorporated. For primary intervention, a monitoring system would help determine whether the overall prevalence of well-being was increasing, and whether there were decreases in the most common or costly behavior problems, including mass shootings. For secondary intervention, monitoring systems have already been developed relative to the specific goals of behavioral activation, ACT, and training on perspective-taking. If implemented for individuals deemed “at-risk,” monitoring systems may be supplemented with additional data on relevant contextual events and behavior patterns such as social isolation, perceived victimization, deficits in perspective-taking, and the existence of behavior traps.

## Conclusion

We believe a behavior-analytic approach can effectively be employed to advance an understanding of mass shooter behavior that enables both prediction and influence. Current nonbehavioral efforts in the area of mass shooting may serve to direct the focus of behavior analysis. As a demonstration, we provided a conceptualized functional analysis that was built on identified behavior–behavior relations. This analysis, in turn, suggested several interventions that may be implemented to influence mass shootings. Although the specific account provided herein focused on fame-seeking mass shooters, the same behavior analytic framework and approach could be applied to other typologies (e.g., workplace shooters) or to other behavioral patterns associated with mass shooters such as misogyny, hate, extremist ideologies, or racism.

It is important to note that the functional account provided in this article is incomplete and additional principles and concepts (e.g., additional rules, habituation to violent content, other categories of relational responding, various establishing operations, basic processes such as respondent conditioning) are undoubtedly involved. In addition, behavior patterns beyond those discussed in this article may be identified. As further behavior patterns and contextual events are identified, additional analysis would be warranted.

A great deal of research on mass shootings has been devoted to categorizing and typologizing mass shooters based on patterns of behavior. Efforts thus far identifying behavior–behavior relations are valuable in establishing warning signs, predicting future shootings, informing risk assessments, and identifying behavior patterns and contextual events that may otherwise remain hidden. Future research should continue to identify additional behaviors and experiences that potentially contribute to mass shootings. This should be done, however, with the understanding that behavior–behavior relations do not explain behavior to an extent that it may be influenced. Behavior may be sufficiently explained through an environment–behavior analysis that identifies manipulable variables in a functional relation with the behavior.

## Data Availability

No new data were created or analyzed in this study. Data sharing is not applicable to this article.

## Footnotes

1 We acknowledge that it is not possible to verify the function of a behavior post-hoc. These actions are not indelible proof that the behavior was motivated by attention but rather are suggestive.
